# Influence of paroxysmal sympathetic hyperactivity (PSH) on the functional outcome of neurological early rehabilitation patients: a case control study

**DOI:** 10.1186/s12883-019-1399-y

**Published:** 2019-07-17

**Authors:** Alan Totikov, Melanie Boltzmann, Simone B. Schmidt, Jens D. Rollnik

**Affiliations:** BDH-Clinic Hessisch Oldendorf, Institute for Neurorehabilitation Research, Associated Institute of the Hannover Medical School, Greitstraße 18-28, 31840 Hessisch Oldendorf, Germany

**Keywords:** Neurological early rehabilitation, Paroxysmal sympathetic hyperactivity (PSH), Outcome, Case control study

## Abstract

**Background:**

Paroxysmal Sympathetic Hyperactivity (PSH) is a frequently observed condition among critically ill patients on intensive care units. According to different studies, PSH is associated with worse recovery and increased mortality in acute-care facilities. In this monocentric, retrospective case-control study, we investigated whether this association also applies to post-acute neurological early rehabilitation.

**Methods:**

The study included *n* = 387 patients, admitted to an intensive care or intermediate care unit within 1 year (2016). Among these, 97 patients showed clinical signs of PSH. For each patient with PSH, a patient without PSH was identified, controlling for age, gender, functional and respiratory status upon admission. However, for 25 patients with PSH, there was no suitable control patient fulfilling all defined matching criteria. Primary outcome was type of discharge, dichotomized into favorable (follow-up rehabilitation) and unfavorable outcome (all others). Secondary outcome measures were functional and respiratory status, number of secondary diagnoses, duration of treatment interruptions and length of stay at discharge.

**Results:**

About 25% of neurological early rehabilitation patients showed clinical signs of PSH. A young age (OR = 0.94; CI = 0.91–0.97) and less severe PSH symptoms (OR = 0.79; CI = 0.69–0.90) were independent predictors of a favorable outcome. In addition, severity of PSH symptoms was associated with weaning duration, while the occurrence of PSH symptoms alone had no influence on most secondary outcome variables. The treatment on intermediate care units proved to be longer for patients with PSH symptoms, only.

**Conclusions:**

Patients with PSH represent a large group of neurological early rehabilitation patients. Overall, we did not find PSH-related differences in most of the examined outcome measures. However, severe PSH symptoms seem to be associated with poorer outcome and longer treatment on intermediate care units, in order to prevent possible complications.

## Background

Paroxysmal Sympathetic Hyperactivity (PSH) is a frequently observed condition during neurological early rehabilitation. Major symptoms include tachycardia, hyperthermia, arterial hypertension, tachypnea, excessive sweating, flexion or extension synergisms with increased muscle tone [1, 2]. Occasionally, further minor symptoms such as myoclonus [1, 2], mydriasis [3], abdominal disorders (constipation or diarrhea) [4], hypersalivation, increased bronchial secretion, hyperactivity, psychomotor agitation [5], blood sugar and metabolic fluctuations [6], as well as flush and goose skin may be observed [2]. Due to this variety of symptoms, various terms have been used to describe this pathology over the last 25 years. Autonomic dysfunction syndrome, autonomic or sympathetic storming, dysautonomia, brainstem attack, hyperpyrexia associated with muscle contraction, hypothalamic-midbrain dysregulation syndrome, acute midbrain syndrome, diencephalic seizure, paroxysmal autonomic instability with dystonia or sympathetic hyperactivity [5, 7, 8] are common terms for PSH. In this paper, the unifying term “Paroxysmal Sympathetic Hyperactivity” is used, which was proposed by an expert consensus group [1].

Currently, the most commonly proposed pathophysiological mechanism causing PSH is a functional disruption or an unbalanced activation of systems subserving autonomic control [9]. Early studies focused on increased diencephalic activity [4, 7, 8], either by direct activation or disinhibition. Most recently, Baguley and colleagues [10] proposed the Excitatory Inhibitory Ratio (EIR) model. This model provides an explanation for the hypersensitive reactions to external stimuli in patients with PSH [9]. Afferents from the spinal cord may disturb the equilibrium through inputs, such as external stimuli [10–12]. The EIR model suggests that the afferent stimuli from the spine have an allodynic tendency (i.e. pain outlasts the external stimulus), which is normally controlled by tonic inhibitory activity of diencephalic structures. A disturbance of these inhibitory structures or of the inhibitory influence on mesencephalic structures could disrupt the control of the allodynic tendency. Once the tonic inhibitory cycle is impaired, there is a positive feedback loop that causes sympathetic overactivity to afferent input [11].

According to several studies, symptoms of PSH occur predominantly among young and male patients with traumatic brain injuries (TBI) (79%) [13, 14]. Although existing publications reported an incidence of PSH between 7.7 and 33% [9], the real incidence of PSH is probably much higher. Baguley and colleagues [11] report that PSH is mainly present within the first week after admission (92%) to the intensive care unit (ICU) and declining during the second week (24%) and beyond that period (8%). However, existing publications do not necessarily reflect the true incidence of PSH.

The aim of the present study is to determine the incidence of PSH in patients during neurological early rehabilitation and to investigate the influence of PSH on the outcome of these patients in dependence of severity of PSH.

## Methods

The study was conducted at the BDH-Clinic Hessisch Oldendorf, a neurological rehabilitation center in Northern Germany with a large intensive care unit (24 beds), three intermediate care units (38 beds) and five peripheral wards (104 beds). The center offers treatment in all phases of neurological rehabilitation under one roof, ranging from acute care treatment (phase A), neurological early rehabilitation (phase B), subsequent rehabilitation (phase C and D) to occupational rehabilitation (phase E).

Neurological early rehabilitation is a specialized treatment for patients suffering from severe neurological disorders of the central and peripheral nervous systems. In the German phase model of neurological rehabilitation [15], neurological early rehabilitation corresponds to phase B, in which intensive care treatment options are still required. Phase B begins as soon as the acute medical treatment (phase A) is over. In case of substantial functional improvements, patients enter subsequent inpatient rehabilitation. When there is no functional gain for a couple of weeks (maximum observation period 2 months), early rehabilitation ends and the patient is discharged to home care or a nursing home.

### Data collection

All neurological early rehabilitation patients admitted to an intensive care or intermediate care unit within 1 year were enrolled in the study (*n* = 387). Each patient was screened for the presence of PSH symptoms using the PSH-Assessment Measure (PSH-AM) proposed by Baguley and colleagues [1], which consists of two subscales. In the Clinical Feature Scale (CFS), specific clinical features of PSH (heart rate, respiratory rate, systolic blood pressure, temperature, sweating and posturing) are examined and assigned 0 to 3 points (see Table 1). Subsequently, the sum of the Clinical Feature Scale for the six features is used to determine a severity score (0 = nil; 1–6 = mild; 7–12 = moderate; ≥13 = severe). The Diagnosis Likelihood Tool (DLT), addressing the specificity of the diagnosis, consists of 11 diagnostic items. The presence of an item is scored as one, while the absence is scored as zero. Finally, the sum of both scores (CFS + DLT) is calculated and used to determine the likelihood of PSH (< 8 = unlikely; 8–16 = possible; ≥17 = probable).

**Table 1:** Clinical Feature Scale [1]

Value	0	1	2	3
Heart rate (bpm)	<100	100–119	120–139	≥140
Respiratory rate (respiration/min)	<18	18–23	24–29	≥30
Systolic blood pressure (mm Hg)	<140	140–159	160–179	≥180
Temperature (°C)	<37	37–37.9	38–38.9	≥39.0
Sweating	nil	mild	moderate	severe
Posturing	nil	mild	moderate	severe

For *n* = 97 patients clinical signs of PSH were identified (25% of all phase B patients admitted to intensive care or intermediate care units). In a next step, control patients without clinical signs of PSH (PSH-) were identified for each patient with PSH symptoms (PSH+). The following criteria were used for the matching process: age (±15 years), gender, Early Rehabilitation Index (±50 points), Barthel Index (±10 points) and mechanical ventilation (yes/no) upon admission. In total, 72 matched pairs were created. Thus, 144 patients were included in the study. For 25 patients with PSH, no suitable control patient fulfilling all defined matching criteria could be found. Especially the combination of low (< 30 years) or high (> 75 years) ages with low functional status upon admission proved to be a difficult match criterion.

Patient data, including demographic data (age, sex) and main diagnosis were retrospectively extracted from electronic patient records. To assess the functional status, the Barthel Index [16] and the Early Rehabilitation Index [17] were determined upon admission and at the end of phase B treatment. The Barthel Index, a measure for activities of daily life, is one of the most common scores in neurological rehabilitation. The functional independence is assessed with ten ordinal-scaled items resulting in a score of 0 to 100 (with 0 being completely dependent and 100 being completely independent from nursing). As an extension to the Barthel Index, the Early Rehabilitation Index addresses aspects important among neurological early rehabilitation patients. In particular, the following criteria are rated: intensive care supervision, tracheostoma, mechanical ventilation, orientation disorder, behavioral disorder endangering oneself or others, severe impairment of communication, and dysphagia. If a criterion is fulfilled, − 25 points (communication impairment) or − 50 points (all other criteria) are assigned (range: − 325 to 0 points).

In addition to these scales, an ICF core set consisting of 20 ICF items was used (see Table 2). The core set was developed and validated by Rollnik [18] as proposal for an ICF-compliant documentation of functional status, as well as for the definition and planning of therapeutic goals in neurological rehabilitation. The severity of each item was scored zero (“no impairment”) to four (“complete impairment”) upon admission and at the end of phase B treatment. ICF data upon admission were only analyzed in *n* = 138 cases when scores from PSH+ and the according PSH- patient were available (*n* = 69 for each group). ICF data at the end of phase B treatment were analyzed in *n* = 50 cases (25 in each group). The main reason for missing endpoint ICF data is that ICF items are only filled out in case of planned discharges. Thus, patients who were transferred to acute hospitals or who died did not have an endpoint ICF assessment. In addition, patients entering subsequent rehabilitation have not been assessed with ICF, either. In these cases, an ICF assessment is done at the end of subsequent rehabilitation (phase C or D) and thus are not comparable to patients at the end of phase B treatment.

**Table 2:** ICF core set proposed by Rollnik [18] for use in neurological rehabilitation

Code	Part 1: Body Functions	Code	Part 2: Activities and Participation
b110	Consciousness functions	d310	Communicating with - receiving - spoken messages
b114	Orientation functions	d330	Speaking
b126	Temperament and personality functions	d440	Fine hand use
b130	Energy and drive functions	d445	Hand and arm use
b140	Attention functions	d450	Walking
b144	Memory functions	d465	Moving around using equipment
b152	Emotional functions	d550	Eating
b156	Perceptual functions	d560	Drinking
b164	Higher-level cognitive functions	d599	Self-care, unspecified
b440	Respiration functions	d850	Remunerative employment

### Outcome measures

For the primary outcome measure, the type of discharge was dichotomized into favorable (follow-up rehabilitation) vs. unfavorable (acute care hospital, nursing at home, nursing home, in-hospital death) outcome. Secondary outcomes included functional improvements (Early Rehabilitation Index, Barthel Index), respiratory status, number of secondary diagnoses (as measure for comorbidity), duration of treatment interruptions as well as length of stay at the end of phase B treatment.

### Statistical analyses

For statistical analyses, the SPSS software package (version 24.0) was used. Since most of the data was not normally distributed, non-parametric statistical methods were used. Group differences were evaluated with the Mann-Whitney U test for metric data and with χ^2^ tests for categorical data. Differences between outcome measures upon admission and at the end of phase B treatment were tested with the non-parametric Wilcoxon test for dependent samples. The Spearman correlation coefficient was used to detect linear relationships. Binary logistic regression models were used to examine which factors predict favorable outcome. All variables available upon admission (age, gender, time since injury, respiratory and functional status and main diagnosis) as well as the score of the clinical feature scale as indicator of PSH severity were tested by forward binary logistic regression for their predictive value of the primary outcome measure (favorable vs. unfavorable outcome).

While categorical variables are presented as percentages, continuous variables are expressed as medians (Md) and interquartile ranges ([IQR], 25th and 75th percentiles). For graphical representations, mean values and standard errors are used. Two-tailed *p* value <.05 was considered significant.

## Results

Baseline characteristics for the total study group and both study groups are presented in Table 3. One hundred forty-four patients with a median age of 58 years (IQR = 49–66) were enrolled in the study (39 female, 105 male). The most frequent main diagnosis was intracerebral hemorrhage (*n* = 40; 27.8%), followed by traumatic brain injury (*n* = 31; 21.5%), stroke (*n* = 25; 17.4%) and hypoxic encephalopathy (*n* = 18; 12.5%). Other diagnoses, such as polyneuropathies, tumors, inflammatory diseases and epilepsy were summarized in the category “other” due to the low number of cases in each category (*n* = 30; 20.8%). The distribution of main diagnoses differed between both groups (χ^2^ = 16.306, *p* = .003). While more patients without PSH symptoms were diagnosed with stroke (χ^2^ = 8.180, *p* = .004) or assigned to the category “other” (χ^2^ = 4.211, *p* = .040), patients with PSH symptoms tended to suffer more frequently from traumatic brain injuries (χ^2^ = 3.330, *p* = .068). The median time from brain injury to admission was 21 days (IQR = 15–29). One hundred twenty patients were admitted to the intensive care unit (83.3%) and 24 patients (16.7%) to an intermediate care unit. There were no differences in terms of age, gender, ventilation and functional status (Early Rehabilitation Index and Barthel Index) upon admission (Table 3).

**Table 3:** Baseline characteristics stratified by PSH group

	Total	PSH+	PSH-	*p* value
Number of subjects	144	72	72	
Age, y (Md; IQR)	58 (49–66)	57 (48–66)	60 (50–67)	.421^a^
Male sex (n; %)	106 (73.6%)	53 (73.6%)	53 (73.6%)	1.000^b^
Tracheal cannula (n; %)	136 (94.4%)	68 (94.4%)	68 (94.4%)	1.000^b^
Ventilated (n; %)	116 (80.6%)	58 (80.6%)	58 (80.6%)	1.000^b^
Time post-injury, d (Md; IQR)	21 (15–29)	19 (14–29)	23 (16–30)	.119^a^
Main diagnosis (n; %)				
Intracerebral hemorrhage	40 (27.8%)	24 (33.3%)	16 (22.2%)	.137^b^
Traumatic brain injury	31 (21.5%)	20 (27.8%)	11 (15.3%)	.068^b^
Stroke	25 (17.4%)	6 (8.3%)	19 (26.4%)	.004^b^
Hypoxic encephalopathy	18 (12.5%)	12 (16.7%)	6 (8.3%)	.131^b^
Other	30 (20.8%)	10 (13.9%)	20 (27.8%)	.040^b^
ERI (median; IQR)	− 150 (− 175;−150)	−150 (−175;-150)	-150 (−175;-150)	.650^a^
BI (median; IQR)	10 (10–10)	10 (10–10)	10 (10–10)	.631^a^

^a^Mann-Whitney U test; ^b^ χ^2^ test. ERI = Early Rehabilitation Index; BI=Barthel Index

### PSH symptoms

Table 4 presents results of the Clinical Feature Scale separately for PSH+ and PSH- group. In the PSH+ group, the median heart rate was 122 bpm (IQR = 102–139), respiratory rate 37/min (IQR = 30–45), systolic blood pressure 178 mmHg (159–197) and temperature 38.1 °C (IQR = 37.4–38.7). The most frequent clinical features were severe tachypnea (*n* = 57; 79.2%) and hypertension (*n* = 34; 47.5%), followed by moderate hyperthermia (*n* = 27; 37.5%) and tachycardia (*n* = 24; 38.3%), excessive sweating (*n* = 26; 36.1%) and severe flexion or extension synergisms with increased muscle tone (*n* = 12; 16.7%). Median values of patients in the PSH- group were significantly different for each category (see Table 4). The following symptoms, which are less frequently described in the literature, were also present in the PSH+ group: abdominal disorders (*n* = 51; 70.8%), electrolyte imbalances (*n* = 49; 68.1%), psychomotor agitation (*n* = 38; 52.8%), hypersalivation (*n* = 35; 48.6%), blood sugar fluctuations (*n* = 24; 33.3%) and myoclonus (*n* = 17; 23.9%).

**Table 4:** Results of the Clinical Feature Scale (CFS) [1] stratified by PSH group

	PSH+	PSH-	*p* value
Heart rate (Md, IQR)	122 (102–139)	103 (89–111)	<.001^a^
Respiratory rate (Md, IQR)	37 (30–45)	21 (16–25)	<.001^a^
Systolic blood pressure (Md, IQR)	178 (159–197)	144 (125–165)	<.001^a^
Temperature (Md, IQR)	38.1 (37.4–38.7)	37.5 (37.1–38.0)	.002^a^
Sweating (n,%)			
1: mild	8 (11.1)	13 (18.1%)	<.001^b^
2: moderate	14 (19.4)	4 (5.6%)	
3: severe	26 (36.1)	–	
Posturing (n,%)			
1: mild	7 (9.7)	7 (9.7)	.002^b^
2: moderate	7 (9.7)	3 (4.2%)	
3: severe	12 (16.7)	–	
Clinical Feature Scale (Md, IQR)	10 (7–13)	5 (3–6)	<.001^a^

^a^Mann-Whitney U test; ^b^ χ^2^ test

The median score of the Clinical Feature Scale was 10 (IQR = 7–13) in the PSH+ group. According to the classification of Baguley and colleagues [2], 16 patients (22.2%) showed mild, 34 patients (47.2%) moderate and 22 patients (30.6%) severe clinical signs of PSH. Table 5 shows the results of the Diagnosis Likelihood Tool. Patients of the PSH+ group exhibited on average eight symptoms (IQR = 6–9). The median sum of the Clinical Feature Scale and the Diagnosis Likelihood Tool was 18 (IQR = 13–22) in the PSH+ group. Thus, 29 PSH+ patients (40.3%) had a “possible” and 43 PSH+ patients (59.7%) a “probable” likelihood of PSH.

**Table 5:** Results of the Diagnosis Likelihood Tool (DLT) [1] for patients with PSH symptoms

Symptom	n (%)
Clinical features occur simultaneously	66 (91.7)
Episodes are paroxysmal in nature	42 (58.3)
Sympathetic over-reactivity to normally non painful stimuli	48 (66.7)
Features persist ≥3 consecutive days	25 (34.7)
Features persist ≥2 weeks post-brain injury	66 (91.7)
Features persist despite treatment of alternative differential diagnosis	49 (68.1)
Medication administered to decrease sympathetic features	70 (97.2)
≥2 episodes daily	27 (37.5)
Absence of parasympathetic features during episodes	29 (40.3)
Absence of other presumed cause of features	50 (69.4)
Antecedent acquired brain injury	66 (91.7)

### Primary outcome measure

The primary outcome parameter of the study was the type of discharge at the end of neurological early rehabilitation, dichotomized into favorable vs. unfavorable outcome. Patients were assigned to the favorable outcome group when they entered subsequent inpatient rehabilitation due to functional improvements (*n* = 33; 22.9%). Patients who died (*n* = 7, 4.9%), those who were transferred to an acute-care hospital (*n* = 21; 14.6%) or a nursing home (*n* = 72; 50.0%) were assigned to unfavorable outcome group. Discharge to nursing at home was also considered as unfavorable outcome (*n* = 11; 7.6%). Thus, *n* = 33 patients (22.9%) were considered to have a positive outcome and *n* = 111 patients (77.1%) to have a negative outcome at the end of phase B treatment. In the whole group, occurrence of PSH symptoms had no influence on the discharge type. In a binary regression model with all variables available upon admission (see Table 3), a low age (OR = 0.94; CI = 0.91–0.97) and a low PSH severity (OR = 0.79; CI = 0.69–0.90) independently predicted a favorable outcome. However, these variables explain 24.5% of the variance of the outcome parameter (*Nagelkerkes R*^*2*^ = 0.245; *p* < .001), only.

### Secondary Outcome Measures

Table 6 presents data of secondary outcome measures for the total study group and both PSH groups. PSH during neurological early rehabilitation was not associated with increased complications, higher comorbidity, and mortality. One hundred thirty-six patients (94.4%) had a tracheal cannula (68 patients in each group) for a median duration of 34 days (IQR = 20–52). Weaning from tracheal cannula was successful in 31 cases (43.1%) in the PSH+ group and in 33 cases (45.8%) of the PSH- group (Z = 0.118; *p* = .731). Mechanical ventilation lasted 280 h on average (IQR = 137–521). The duration of mechanical ventilation was influenced by the severity of PSH symptoms (Z = 6.685, *p* = .035). In detail, mild PSH symptoms (Md = 136 h; IQR = 109–285) were associated with a shorter ventilation period than moderate (Md = 308 h; IQR = 138–567; Z = -2.060, *p* = .038) and severe (Md = 369 days; IQR = 231–602; Z = -2.630, *p* = .007) PSH symptoms. Weaning from mechanical ventilation was successful in 90 cases (80.6%). The longer patients were dependent on mechanical ventilation, the longer they stayed on ICU (r = 0.851, *p* < .001). This relationship, however, was influenced by age: A younger age was associated with a shorter ventilation period (r = 0.246, *p* = .003), which in turn shortened the length of stay on ICU (r = 0.200; *p* = .016).

**Table 6:** Secondary outcome measures stratified by PSH group

	Total	PSH+	PSH-	*p* value
Number of subjects	144	72	72	
Duration of tracheal cannula, d (Md; IQR)	34 (20–52)	35 (25–49)	27 (14–55)	.182^a^
Duration of ventilation, h (Md; IQR)	280 (137–521)	296 (131–519)	268 (139–667)	.947^a^
Successful weaning (n; %)	90 (62.5%)	45 (62.5%)	45 (62.5%)	1.000^b^
Secondary diagnoses, n (Md; IQR)	32 (27–36)	33 (27–35)	32 (25–37)	.792^a^
Duration of interruptions, d (Md; IQR)	7 (5–14)	7 (5–13)	7 (4–15)	.841^a^
Length of stay, d (Md; IQR)	75 (46–108)	77 (56–110)	73 (42–103)	.303^a^
ΔERI, points (Md; IQR)	100 (50–150)	100 (50–150)	75 (25–150)	.988^a^
ΔBI, points (Md; IQR)	5 (5–22.5)	5 (5–10)	5 (5–28)	.113^a^

^a^Mann-Whitney U test; ^b^ χ^2^ test. ERI = Early Rehabilitation Index; BI=Barthel Index

In total, patients were treated for 75 days (IQR = 46–108) in phase B rehabilitation. A detailed analysis separately for the different wards revealed that PSH symptoms were associated with prolonged length of stays on intermediate care units (Z = -2.083; *p* = .037). While most patients were treated continuously (*n* = 89; 61.8%), 55 patients (38.2%) had at least one interruption (one interruption: *n* = 41; two interruptions: *n* = 12; three interruptions: n = 1). Median duration of all interruptions was 7 days (IQR = 5–14). In all cases, patients were transferred to acute-care hospitals. Main reasons were implantations of shunts or drug pumps, replacement of bone flap (skull), wound management or treatment of other complications.

**Fig. 1:**
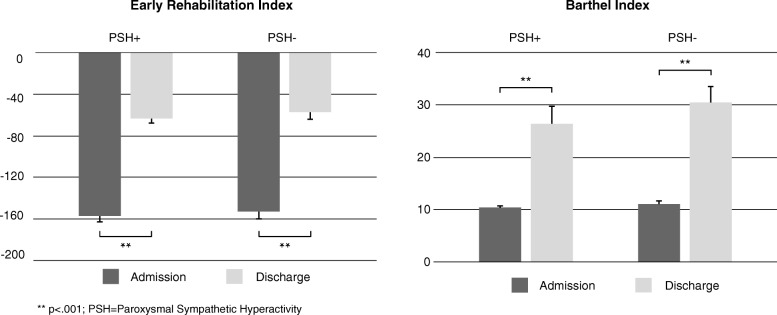
Improvements in functional measures during phase B treatment

**Fig. 2:**
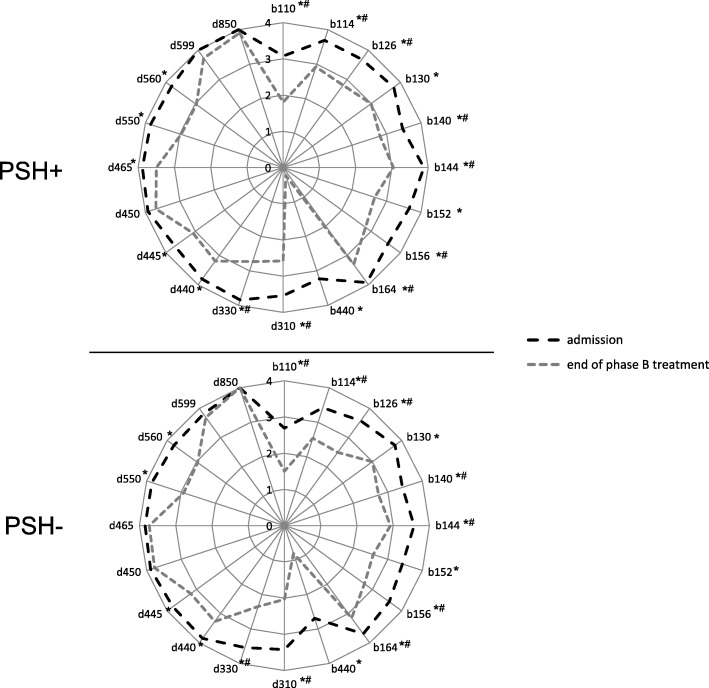
ICF assessment of both study groups upon admission (*n* = 138) and at the end of phase B treatment (*n* = 50). * Wilcoxon test for paired samples, *p*-value<.05; ^#^ Mann-Whitney U test, *p*-value<.05

Figure 1 shows that the functional status of both groups (PSH+/PSH-) improved during phase B treatment. Group differences, however, were not observed at the end of phase B treatment. Due to these results, ICF data were studied for a deeper analysis of the course of rehabilitation, although they were not used as criteria for the formation of matched pairs. Unfortunately, patients with PSH had significantly worse scores in about half of all items upon admission (Fig. 2). However, both groups showed significant improvements in most variables. At the end of phase B treatment, no group differences were observed anymore.

### Subgroup analysis for patients with severe PSH symptoms

**Fig. 3:**
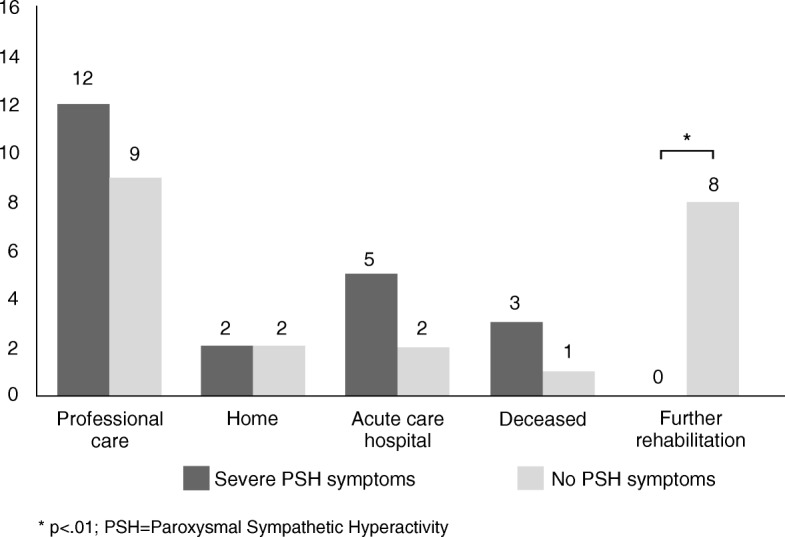
Frequencies of discharge type, presented for patients with severe PSH and control patients

A severity-related analysis revealed that patients with severe PSH symptoms (*n* = 22) were more likely to have an unfavorable outcome compared to control patients (χ^2^ = 9.778, p = .002). This was due to the fact that few control patients (n = 8) entered subsequent rehabilitation (Fig. 3). In addition, patients with severe symptoms of PSH were treated significantly longer on intermediate care units (54 vs. 26 days; Z = -2.009; *p* = .045) and showed less progress in functional measures (Barthel Index: 5 vs. 22 points; Z = -2.548, *p* = .016) than controls. Time until withdrawal of tracheal cannula was longer for patients with severe PSH symptoms (59 days) than patients with moderate PSH symptoms (34 days; Z = -2.101, *p* = .036). The severity of symptoms was also associated with age (Z = 10.789, *p* = .005). Patients with severe PSH symptoms were younger (48 years) than patients with mild (56 years; Z = -2.485, *p* = .012) and moderate (60 years; Z = -3.097, *p* = .002) symptoms. Other measures upon admission (gender, main diagnosis, ventilation, time post-injury, functional status) were not modulated by the severity of PSH symptoms.

## Discussion

The aim of this study was to investigate the influence of PSH symptoms on different outcome measures and whether this association is influenced by the severity of PSH symptoms. For this purpose, patients with and without PSH symptoms were studied in a matched case-control design. The prevalence of PSH among all patients enrolled in the study was 25% (*n* = 97). However, in 72 cases, only, a suitable control subject fulfilling all matching criteria could be identified. In this subgroup, a lower age and a lower PSH severity independently predicted the primary outcome measure (favorable vs. unfavorable outcome). A favorable outcome was defined as entering subsequent in-house rehabilitation, while an unfavorable outcome comprised long-term nursing care, transfer to acute-care hospitals or in-hospital deaths. With respect to secondary outcome variables, length of stay on intermediate care units only proved to be longer for patients with clinical signs of PSH. In contrast, length of stay on the intensive care unit and peripheral wards were not associated with the presence or severity of PSH. This result is in line with the study of Baguley and colleagues [2]. The study showed that patients with PSH symptoms had significantly longer rehabilitation, while length of stay on intensive care units was not different. Fernández-Ortega and colleagues [6] report longer stay on intensive care units of patients suffering from acute dysautonomic crises. Comparing these studies, however, should be interpreted carefully due to different study designs (e.g., acute vs. post-acute setting; traumatic only vs. traumatic and non-traumatic etiologies).

Severity-related analyses revealed that patients with severe PSH symptoms were more likely to have an unfavorable outcome than control patients. This finding was mainly caused by better improvements among controls (Barthel Index) allowing them to enter subsequent in-house rehabilitation. In contrast, none of severe PSH patients entered subsequent rehabilitation. Previous studies demonstrated that PSH is associated with a worse functional outcome compared to patients without PSH symptoms. For example, PSH patients showed worse scores on the Glasgow Coma Scale compared to non-PSH patients at the end of treatment [2, 6, 19]. This finding is in line with the results of the present study, because a worse functional outcome was only observed in severe PSH- patients. Although the results suggest that patients with severe PSH symptoms did not benefit from inpatient rehabilitation as much as patients with mild and moderate PSH symptoms, several other variables (i.e., etiology, location of brain damage) and thus the level of consciousness and impairments (cognitive-, motoric-, speech-) have an impact on outcome. The current study used the Barthel Index and Early Rehabilitation Index upon admission as match criteria to homogenize the functional status of both groups at study entry. However, a recent study [20] investigating clinical factors influencing the weaning of phase B patients, showed that successfully and non-successfully weaned patients did not differ in Early Rehabilitation Index, but in other assessment scales (i.e. the Glasgow Coma Scale and Functional Ambulation Categories) upon admission. This suggests that functional status might be better evaluated by other assessment scores, which was also confirmed by the fact that ICF scores differed between both groups upon admission, although Barthel Index and Early Rehabilitation Index did not (both were used as match criteria).

To reliably detect, monitor and treat PSH symptoms it is recommended to implement diagnostic tools (e.g., PSH-AM) in early rehabilitation settings. Effective treatment, however, can be challenging. A clear association between specific symptoms and drug selection may help to avoid over−/underdosing and side effects, decline the clinical status and hamper improvements during early rehabilitation. A combination of drugs from different classes [9] with rehabilitation interventions is supposed to be most effective. Most PSH symptoms occur as an allodynic response to external stimuli, such as pain, urinary retention, or movements. When such triggers may be identified, it is reasonable to either treat or to avoid them. Opioids, morphine, and other sedatives (e.g. midazolam) are used as first-line drugs to suppress the allodynic response in PSH patients [21]. Non-pharmacologically, the avoidance of external stimulation and maintaining comfortable room temperatures are of major importance.

Another important issue was to examine whether the presence of PSH has an impact on the weaning process (i.e. withdrawal from mechanical ventilation). The analysis revealed that the duration of mechanical ventilation was not related to PSH. The median weaning duration is consistent with the results of previous studies investigating the outcome of neurological early rehabilitation patients [22, 23]. A currently published guideline on weaning [24] has not reported any specific effect of PSH on the weaning process. In contrast, the present study showed an influence of PSH severity on weaning duration. However, several other factors such as the need of dialysis or colonization with multi-drug-resistant bacteria are associated with weaning failure during phase B treatment [20]. Additionally, the presence of PSH symptoms and PSH severity had no influence on length of stay at the ICU. This result supports the finding that weaning duration was comparable in both groups, since patients are usually transferred to an intermediate care unit as soon as they are successfully weaned from mechanical ventilation. In contrast to ICU, however, patients with PSH symptoms were treated longer on intermediate care units than control patients. This relationship particularly applied to patients with severe PSH symptoms. Most likely, these patients stayed longer on intermediate care units because they needed more time for the withdrawal of the tracheal cannula.

### Limitations

Comparisons of the present results to international studies are difficult [25]. One reason is that neurological early rehabilitation is a special treatment for patients with severe brain injuries in Germany. In addition, international studies frequently focus on one etiology, only (e.g., stroke, traumatic brain injury or intracerebral hemorrhage). However, the current study aimed to predict outcome of early rehabilitation patients consecutively admitted to intensive care or intermediate care units, irrespective of main diagnosis. Unfortunately, this approach resulted in a different distribution of main diagnoses for both study groups (PSH+/PSH-). Each main diagnosis has an impact on the rehabilitation process and may influence PSH symptoms in an individual manner, potentially biasing the results. Thus, the main diagnosis should be used as match criterion in future studies. Nevertheless, since we used a multivariate binary regression model controlling for potential effects of other variables (e.g., main diagnosis), age and PSH severity may be considered as independent predictor of favorable outcome.

In addition, in 25 PSH cases, no suitable controls were available fulfilling all matching criteria. Thus, the true number of patients with clinical signs of PSH was limited due to the study design. Since the combination of low or high age and low functional status upon admission proved to be a difficult match criterion, especially patients with severe functional impairments had to be excluded from the study. This might be important for the present results, as this group tends to be most adversely affected by PSH symptoms.

## Conclusion

The study contributes knowledge showing that the prevalence of PSH in neurological early rehabilitation is similar to acute-care settings. In summary, this study revealed that the occurrence of PSH symptoms was not associated with more complications, higher comorbidity or mortality. PSH-related differences were observed for length of stay on intermediate care units, only. Patients with severe PSH symptoms, however, were more likely to suffer from worse outcome compared to control patients. These results are significant for the diagnosis, monitoring and treatment of PSH symptoms.

## Data Availability

The datasets used during the current study are available from the corresponding author on reasonable request.

## Abbreviations

ICF: International Classification of Functioning, Disability, and Health
ICU: Intensive Care Unit
PSH: Paroxysmal Sympathetic Hyperactivity
PSH-AM: PSH-Assessment Measure
TBI: Traumatic Brain Injury
